# Design and performance of a capacitor-based burn-through monitor for high-power X-ray beams at XFEL facilities

**DOI:** 10.1107/S1600577526008647

**Published:** 2026-09-16

**Authors:** Willem Langeveld, Michael Rowen, Alyssa Prinz, Rebecca Armenta, Matthieu Chollet, Carolyn Gee, Phil Heimann, Josh Kirks, Kazutaka Nakahara, Eliazar Ortiz, Sayed Rokni, Andrew Rosenstrom, Takahiro Sato, Bill Schlotter, Brian Smith, Claudia Stefanescu, Ydran Willard, Shanjie Xiao, Hasan Yavas, Diling Zhu

**Affiliations:** a SLAC National Accelerator Laboratory, Linac Coherent Light Source, 2575 Sand Hill Road, Menlo Park, CA94025, USA; European XFEL, Germany

**Keywords:** X-ray burn-through monitors, X-ray beamline diagnostics, beamline containment, personnel protection

## Abstract

The reliable and rapid detection of uncontrolled X-ray beam propagation is critical for machine and personnel protection at high-power X-ray free-electron lasers. We present the design, operation, and performance of the X-Ray Flux Capacitor (XRFC) burn-through monitor (BTM), a parallel-plate capacitor device implemented as a printed circuit board, intended to provide an inexpensive, scalable, and fail-safe beam containment monitoring solution when used with appropriate readout electronics. The capacitor BTM operates on the principle that focused high-power X-rays degrade the FR-4 dielectric between the capacitor layers, resulting in a high-voltage electrical short that is easily detectable. Ninety-two high-voltage prototype capacitor BTMs were tested at various beam parameters at the Linac Coherent Light Source XCS and XPP endstations, using X-ray energies between 7.17 keV and 13.1 keV. All capacitor BTMs shorted when exposed to beam conditions with sufficient energy density, with shorting thresholds ranging from 0.7 eV atom^−1^ pulse^−1^ to 5.8 eV atom^−1^ pulse^−1^ (in copper), depending on X-ray energy and beam focus. In all cases, a high-voltage short was detected before full burn-through of the capacitor BTM occurred. These results suggest that the XRFC BTM concept can be used as a reliable, fast-response diagnostic for beam integrity monitoring in high-flux environments.

## Abstract 2

We present the results of testing 92 prototypes of a capacitor-based burn-through monitor at the LCLS XCS and XPP endstations. We quantify the beam energy density thresholds required to induce a detectable high-voltage electrical short prior to complete burn-through and demonstrate the reliability of this scalable, low-cost solution for machine and personnel protection at high-power light sources.

## 1. Introduction

High-power X-ray free-electron lasers (XFELs), such as the Linac Coherent Light Source (LCLS), generate intense, femtosecond-duration X-ray pulses capable of delivering high energy densities (LCLS, 2002 ▸; LCLS-II, 2015 ▸; Schoenlein *et al.*, 2016 ▸; LCLS-II, 2024 ▸). While these characteristics enable groundbreaking experiments in physics, chemistry, and biology, they also present significant challenges to machine and beamline integrity as well as personnel protection. An uncontrolled or misaligned beam, particularly when focused, can rapidly damage critical components, including diagnostics, beam transport optics, vacuum hardware, beam stoppers, collimators and beam dumps, possibly allowing the X-ray beam to penetrate walls and endangering personnel. Therefore, the development and implementation of reliable and fast-acting beam monitoring systems are essential for machine and personnel protection.

In the past, absorber materials for X-ray beam containment devices, such as photon beam stoppers, photon collimators and photon dumps, were designed with the capability to passively absorb the X-ray beam power. With the advent of newer, more powerful XFEL facilities, this is no longer sufficient (LCLS-II, 2024 ▸). Therefore, robust and fail-safe burn-through monitors (BTMs) are essential to detect beam conditions that could lead to absorber material failure. Upon detection of such conditions, a BTM must provide a trip signal to the beam containment system (BCS) and/or personnel protection system (PPS), which in turn will result in shutting the X-ray beam off. An ideal BTM must possess several characteristics: it must be highly sensitive to signs of material damage, possess a response time fast enough to permit beam shut-off before safety shielding is breached or perforated (preferentially within one XFEL pulse, but at least significantly faster than it takes to burn through the last absorber element of the safety device), and have a reliable way to distinguish beam-caused trips from nuisance trips after the fact. Ideally, the device should be inexpensive and scalable for large-area coverage and offer simple and reliable read-out. Existing solutions, such as air-bladder BTMs (Mancuso *et al.*, 2013 ▸; Madsen *et al.*, 2013 ▸) and scintillator or fluorescence-based BTMs (Clement, 2023 ▸; Dommach, 2023 ▸) often fall short of meeting all these criteria simultaneously.

The X-Ray Flux Capacitor (XRFC) burn-through monitor, referred to as the capacitor BTM for short, was developed to address these requirements. The concept involves a parallel-plate capacitor implemented as a multi-layer printed circuit board (PCB). A voltage is applied to one set of layers, and the other (interleaved) layers are kept at ground potential. The initial design hypothesis centered on the formation of a low-resistance plasma channel upon burn-through, allowing the capacitor to discharge and provide a measurable voltage drop.

Subsequent analysis, however, suggested that plasma conduction alone might not generate a sufficient signal, particularly at low operating voltages. This led to an adapted design focused on high-voltage operation, based on the principle that the intense X-ray pulse would degrade the FR-4 dielectric material between the copper layers, inducing a high-voltage electrical short. This (usually permanent) short provides an unambiguous and measurable signal that can be used to shut off the beam. Additionally, the high-voltage arc leaves behind a clear mark on the device, allowing for verification that a real burn-through occurred after the fact.

This paper reports on experimental tests of capacitor BTM prototypes in an XFEL beam at atmospheric conditions. As expected, initial low-voltage prototypes showed poor performance, but high-voltage prototypes tested in early 2024 at the X-Ray Correlation Spectroscopy (XCS) instrument demonstrated excellent reliability. Based on these results, improved prototypes of various designs and sizes with PCBs manufactured by four different vendors were tested at the XCS and the X-Ray Pump Probe (XPP) instruments in late 2024.

Based on 92 tested prototypes, this work aims to establish the performance characteristics, energy density thresholds, and reliability of the high-voltage capacitor BTM concept as a viable machine- and personnel-protection diagnostic for XFEL facilities.

## 2. Experimental methods

### 2.1. Capacitor BTM design and manufacturing

The capacitor BTM is fundamentally a parallel-plate capacitor in the form of a multi-layer printed circuit board. The basic electrical circuit is shown in Fig. 1 ▸. High voltage (*V*_0_) is applied to one side of a capacitor through a 4 MΩ resistor. The capacitor voltage is monitored via a resistive divider network (100 MΩ and 100 kΩ) providing a 1/1000 nominal voltage read-out: for typical operation at 1000 V, the monitor output is approximately 1 V. Details, including the physical stack-up, are provided in Appendix *A*1[App appa]. The prototypes have ten layers of 1-ounce copper (35 µm thick) separated by FR-4 dielectric material. Five copper layers are connected to high voltage (HV) and are interleaved with the other five layers at ground potential. The separation between any HV layer and the closest adjacent ground layer is small enough that the nominal HV cannot be sustained in air without the presence of intact FR-4 dielectric material. If the dielectric material is sufficiently damaged, an arc and subsequent short is unavoidable. In rare cases, the arc can burn away some of the copper surrounding the location of the short, leading to ‘self-healing’, in which case the device can partially or fully recover the original high resistance between HV and ground layers, causing the monitor output voltage to return to its initial value. However, a clear and long-duration signal is detected even during such temporary shorts. The minimum signal duration is determined by the product of the 4 MΩ resistor value and the capacitance of the device.

A total of 92 high-voltage capacitor BTM prototypes were tested in 2024, including 19 original prototypes and 73 improved prototypes of varying sizes. The PCBs were sourced from four different manufacturers [Vector Fabrication (https://vectorfab.com/), JLCPCB (https://jlcpcb.com/), PCB­Way (https://www.pcbway.com/), and Cirexx (https://www.cirexx.com/)] to investigate the dependence of performance on fabrication consistency. Four active area sizes were tested:

(i) Original: 100 mm × 85 mm (capacitance 25–27 nF), see Fig. 2 ▸(*a*).

(ii) Small, annular area: 75 mm outer and 25 mm inner diameter (capacitance 15–17 nF), see Fig. 2 ▸(*c*).

(iii) Medium: 195 mm × 175 mm (capacitance 87–103 nF), see Fig. 2 ▸(*b*).

(iv) Large: 625 mm × 460 mm (capacitance 750–850 nF), see Fig. 2 ▸(*d*).

For further details, see Appendix *A*[App appa].

### 2.2. Experimental setup and data acquisition

#### 2.2.1. Capacitor BTM mounting and power

Prototypes were housed in custom-designed acrylic enclosures with an SHV connector for high-voltage input and a BNC connector for the monitor output signal. A Canberra model 3002D high-voltage power supply was used in the tests. A mechanical safety interlock required the removal of the high-voltage cable before the door could be opened.

The enclosures had holes in the front to allow the X-ray beam to enter unimpeded. Following initial tests between 500 V and 2000 V, all subsequent experiments were conducted at a nominal operating voltage of 1000 V, well below the maximum sustainable voltage of the PCB. The latter was determined using an additional original prototype by increasing the high voltage until a spontaneous short occurred. That unit failed at ∼2500 V, even though it was not designed for high voltage. Recent testing has shown that the newer prototypes can withstand voltages of at least 4550 V. A manuscript detailing extensive high voltage endurance testing is in preparation.

The capacitor BTM enclosures were positioned at the interaction point (IP) in the focal plane of the XFEL beam at the XCS and XPP endstations. A capacitor BTM prototype was inserted into the enclosure before each test and replaced with another one after the measurement.

#### 2.2.2. Diagnostics and read-out

Photodiodes (at XCS) or PIPS diodes (at XPP) were used upstream of the enclosure to monitor scattered X-rays from the capacitor BTM, and downstream to detect any transmitted X-rays. The downstream diode was positioned to detect X-ray scattering from a sacrificial Kapton film or steel plate placed in the path of the beam behind the capacitor BTM. A schematic is shown in Fig. 3 ▸.

For the newer prototypes, three simultaneous data streams were collected to capture both the fast transient electrical short and the long-term voltage status. A Siglent SDS3054-X HD oscilloscope (500 MHz, 12 bit) recorded the capacitor BTM monitor output and the photodiode signals at 50 MSPS for up to 1 s duration. The trigger was set to capture any drop in the capacitor BTM signal below 75% of the nominal value. A Texas Instruments ADS1256 (24 bit) ‘slow’ ADC connected to a microcontroller continuously recorded the monitor output signal at 500 samples per second to track the long-term capacitor voltage stability and the permanent (or temporary) state change. The capacitor BTM signal, along with photodiode and/or PIPS diode signals, was further connected to the facility’s data acquisition system. A SLAC Wave8 gen-1 digitizer (at XCS) or an Acqiris digitizer (at XPP) captured waveforms for pulse-by-pulse analysis.

## 3. Results and discussion

A total of 92 capacitor BTM prototypes were tested with the LCLS XCS and XPP instruments in 2024. The original 19 prototypes were tested for shorting when struck by a sufficiently intense beam, and, while all of them shorted, insufficient data were collected for these units to be included in the rest of this study. Some data for four of the newer prototypes tested at XCS were lost, and while those units also shorted, they were not used in the following analysis. This leaves the 29 newer prototypes tested at XPP and 40 newer prototypes tested at XCS.

Attenuators upstream of the interaction point (IP) were used to vary the beam pulse energy. For focused beam, a compound refractive lens (CRL) stack was inserted. Testing spanned multiple X-ray energies and beam conditions, with both focused and defocused (CRL inserted but moved out of focus) configurations. With unfocused beam no shorts or burn-throughs were detected, and no clear burn marks were observed. Table 1 ▸ shows a summary of the beam parameters used. Since no shorts or burn-throughs occurred with unfocused beam, Table 1 ▸ only shows the typical unattenuated pulse energy at the interaction point for focused beam, *i.e.* with the focusing lens stack inserted. The upstream attenuators were used to reduce the pulse energy further in most runs in order to establish the minimum required energy density to cause a short or burn-through.

### 3.1. Energy density calculation

In the following, the data from the experiments are plotted as a function of the energy density of the beam striking the BTM. The energy density ɛ at the IP is given by
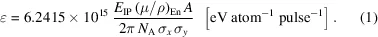
Here, *E*_IP_ is the beam pulse energy at the IP in mJ/pulse, (μ/ρ)_En_ the mass-energy absorption coefficient of the material struck by the beam in cm^2^ g^−1^, *A* the atomic mass in g mol^−1^, *N*_A_ Avogadro’s constant, and σ_*x*_ and σ_*y*_ are the standard deviations of the (assumed Gaussian) beam profile in the *x* and *y* directions in cm. The factor in front converts mJ to eV (1/*e*/1000, with *e* the electron charge). The pulse energy at the IP, *E*_IP_, was computed from the pulse energy measured by a gas detector in the front-end enclosure (FEE), any attenuators inserted in the beam downstream of the gas detector, and the inherent attenuation caused by the focusing lens stack (if inserted). For (μ/ρ)_En_ the values for copper were used, since that is the first significant material encountered by the X-ray beam when it strikes the capacitor BTM.

There is a significant systematic uncertainty in the energy density because the quantities σ_*x*_ and σ_*y*_ at the focal point are difficult to measure due to the small (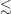
3 µm FWHM) spot sizes achievable at LCLS beamlines. Note that this uncertainty enters quadratically in the equation. It is difficult to quantify this uncertainty, especially near the low energy densities required for a short. From the data, we estimate the uncertainty in the energy density to be roughly 30% near 1 eV atom^−1^ pulse^−1^. We do not think that this uncertainty affects the analysis.

In this work, the beam spot sizes were estimated from CRL *z*-scans (moving the CRL stack along the beam axis) with the beam striking a YAG screen located at the IP observed by a camera, resulting in an *x*–*y* image. The pixel size was calibrated using images from unfocused beam passing through a set of horizontal and vertical slits of known width. Using this calibration, the *x* and *y* spot sizes were obtained from the image using Gaussian fits and plotted versus the *z*-position of the CRL stack. The resulting curve is a quadratic sum of the actual beam spot size and the size of the point-spread function (PSF) of the measurement setup, where the PSF is the image that would be produced by a hypothetical point source of beam. The PSF was estimated by a fitting procedure that combines a PSF contribution with the ideal focused Gaussian beam propagation profile appropriate to the X-ray energy. The actual beam spot size at any CRL *z* location was then recovered by deconvolving the measured curve with the PSF. For a full description of the procedure, see Langeveld (2025 ▸).

During the experiments, the CRL stack was moved along *z* to obtain focused or defocused beams.

### 3.2. Analysis of data taken at XPP

The XPP experiments were conducted at 10 keV X-ray energy. Fig. 4 ▸ shows the cumulative deposited energy in the device until a short occurred versus the X-ray energy density at the device for 29 XPP measurements.

The lowest energy density causing a short was approximately 1.3 eV atom^−1^ pulse^−1^. Capacitor BTMs in full focus (∼2.9 µm beam diameter FWHM) shorted consistently at energy densities above 2 eV atom^−1^ pulse^−1^; those in defocused beam (∼17 µm) shorted at 1–2 eV atom^−1^ pulse^−1^, though often requiring many more beam pulses (higher cumulative deposited energy) before the short occurred. The total cumulative energy required to induce a short varied significantly across attenuation levels but showed a clear dependence on energy density, as plotted in Fig. 4 ▸.

A short-duration voltage ‘blip’ lasting approximately 140 ns was observed in the oscilloscope trace preceding the actual high-voltage short in one run, see Fig. 5 ▸-left. No similar transient signals were detected in any other run where an oscilloscope was set up to trigger on such events (68 runs). The oscilloscope signal from a more typical run is shown in Fig. 5 ▸-right—note the difference in the horizontal scale (ns versus µs).

The origin of this blip remains unclear, potentially stemming from electronic noise or a transient physical effect within the capacitor BTM. The capacitor BTM was under HV and there was X-ray beam on the device at the time, so this could be deemed a legitimate trip in that sense, since in an operational scenario the beam should never be allowed to reach the BTM. Because the unit shorted permanently soon afterwards leaving a distinct mark, it is not known whether a visible mark would have resulted from just this transient event. This is discussed further below.

Fig. 6 ▸ shows traces captured by the slow ADC. Notice the different horizontal scale (seconds). The left trace is of a run where the capacitor shorted outright. The center trace is of the run with the blip, which was not detected by the slow ADC. Instead, the capacitor shorted soon after, and, although it tried to recover several times, it eventually shorted permanently. The trace on the right is of a run where the capacitor BTM recovered after the short. Further details about this type of event are given later.

### 3.3. Analysis of data taken at XCS

The XCS experiments provided data at three different X-ray energies (7.17, 9.99 and 13.1 keV). Fig. 7 ▸ shows the cumulative deposited energy in the device until a short occurred versus the energy density at the device for 40 XCS measurements. As mentioned, four runs were omitted from this analysis.

Shorting thresholds varied with beam energy and spot size (see Table 1 ▸). The lowest observed shorting thresholds at XCS were 0.7 eV atom^−1^ pulse^−1^ at 7.17 keV, 5.8 eV atom^−1^ pulse^−1^ at 9.99 keV and about 2 eV atom^−1^ pulse^−1^ at 13.1 keV. This variation shows that the damage mechanism depends on X-ray energy, which is not surprising given that penetration depth and interaction cross-section of the X-rays are functions of energy.

Near the threshold, where the energy density is barely sufficient to cause any damage at all, run-to-run variations are sufficient to significantly affect the required number of pulses, and thus the cumulative deposited energy, for a short to occur. No correlations between vendors or sizes or formats were found.

### 3.4. Mechanism

The results presented here are consistent with the following physical mechanism (Langeveld *et al.*, 2025 ▸). At high beam energy density, a single pulse may be sufficient to drill a hole through the first copper layer and degrade, carbonize or remove the FR-4 between layers, causing an arc between the copper layers. At low beam energy density, multiple pulses are usually required to heat and degrade the FR-4 between layers sufficiently so that cumulative damage will cause an arc to occur between the copper layers. While in principle the beam energy density can be below the copper damage threshold, provided that the FR-4 between the first copper layers heats up sufficiently, the measured minimum energy densities required for a short are generally above the copper damage threshold (∼0.4 eV atom^−1^ for melting, ∼0.9 eV atom^−1^ for boiling).

Beam-pulse by beam-pulse analysis of the data has shown that the short occurs before full burn-through of the entire device. This shows that only the first few layers are involved in the short. Since the FR-4 thickness between the second and third copper layers is larger than that between the first and second copper layers, it is likely that the short occurs between the first and second copper layers. Notably, the visible mark left by the short is always on the side of the device facing the beam. But because of the damage caused by the arc, it is not possible to determine whether more than one FR-4 layer is involved.

### 3.5. Overall capacitor BTM performance

The fundamental operational goal of the capacitor BTM, *i.e.* to fail with a detectable electrical short prior to complete capacitor BTM burn-through, was consistently met across all tested prototypes.

In all experiments conducted at sufficient energy density, the capacitor BTMs exhibited a high-voltage short. This short caused a rapid drop in the monitor output voltage, which triggered the oscilloscope and the DAQ system. In eight out of 64 runs where long-duration slow ADC traces were recorded, the capacitor BTM shorted only temporarily (‘self-healed’). An example of a slow ADC trace captured in such a run is shown in Fig. 6 ▸-right. In these runs, the beam was used in burst mode, causing it to be shut off rapidly after the short. In other runs where burst mode was used, the capacitor BTM shorted permanently. But in many runs the beam continued even after a short was detected, and if the beam had been shut off immediately it is quite possible that more self-healing events would have occurred. Note that the blip event mentioned previously, even though it was transient, was not counted as a self-healing event. The blip did not exhibit the characteristics of a short: a fast signal drop to zero (and below, see Fig. 5 ▸-center) and a slow recovery (time scale of about 1 s, see Fig. 6 ▸-right). Instead, the blip duration was only 140 ns (see Fig. 5 ▸-left), and a full short did not occur.

We note that self-healing events are not false alarms and are not considered problematic: the initial short of the device results in an unmistakable fast and long-duration detection signal, which, together with latching electronics, can be used to provide fail-safe operation in the same manner as is done with other types of BTM. This is discussed in more detail in Appendix *A*2[App appa]. After any shorting event the device should, however, be considered damaged and in need of replacement; whereas self-healed devices have been seen to short again when exposed to X-ray beam, this has not been extensively tested.

For every short event, including in cases of self-healing, an easily visible mark was left on the front PCB surface, see for example Figs. 8 ▸ and 9 ▸. The size of the mark was observed to correlate with the stored energy, with shorts in smaller (lower capacity) capacitor BTMs resulting in smaller marks, suggesting that adding supplementary capacitance could make the failure sign more conspicuous for small BTMs.

Critically, in no instance did a capacitor suffer a full burn-through without first registering an electrical short. Analysis of the pulse-by-pulse data confirmed that, if the XFEL beam were shut off before arrival of the beam pulse following the detection of the electrical short, burn-through of the entire capacitor BTM would have been prevented in all tested prototypes.

No significant differences in performance or failure threshold were observed between capacitor BTMs fabricated by the four different PCB vendors. Taken together, these results validate the suitability of the capacitor BTM for incorporation into a fast machine and personnel protection system.

### 3.6. Future considerations

The observed ‘blips’ and ‘self-healing’ events suggest further work is required. The potential for electronic noise or spontaneous shorts necessitates long-duration high-voltage testing to investigate potential ‘burn-in’ requirements and the likelihood of false trips. Recently, a study of the voltage endurance of capacitor BTMs has been completed and a follow-on publication is in preparation. The general conclusion is that capacitor BTMs are capable of sustaining 1000 V for many years, but a burn-in screen for units with manufacturing defects is advised.

Whereas we do not expect the capacitor BTM to be very sensitive to radiation because of its simple design, we have not tested the device in a high radiation environment. In case of deployment in such an environment, additional tests should be conducted. Also, if operation in a vacuum is anticipated, this should be tested.

Future designs may also incorporate an additional capacitor to increase the energy released upon shorting, making the visual failure mark, particularly on small BTMs, more apparent.

## 4. Conclusions

Extensive testing using 92 high-voltage capacitor BTM prototypes strongly suggests that the capacitor BTM is reliable and suitable for a machine and personnel protection diagnostic at high-power XFEL facilities, when paired with suitable readout electronics.

(i) All BTMs shorted under exposure to sufficient X-ray energy density, suggesting that X-ray-induced degradation of the FR-4 dielectric is a reliable failure mechanism. For a more detailed discussion of the FR-4 failure mechanism, see Langeveld *et al.* (2025 ▸). No false trips were detected.

(ii) Analysis of event-by-event data shows that a high-voltage short is detected prior to full burn-through of the capacitor BTM itself in all tested cases, and the signal is in principle fast enough to shut off the beam before the next pulse. Because it may take some time to shut off the beam, additional time can be gained by putting an absorber behind the capacitor BTM with a known burn-through time for the expected worst-case beam conditions.

(iii) In all cases, a clear burn mark was found on the face of the capacitor BTM, demonstrating verifiability of actual burn-through, in contrast to false (‘nuisance’) trips.

(iv) Performance was found to be independent of the manufacturing vendor or device size (within the tested range), indicating a robust and scalable design.

## Figures and Tables

**Figure 1:**
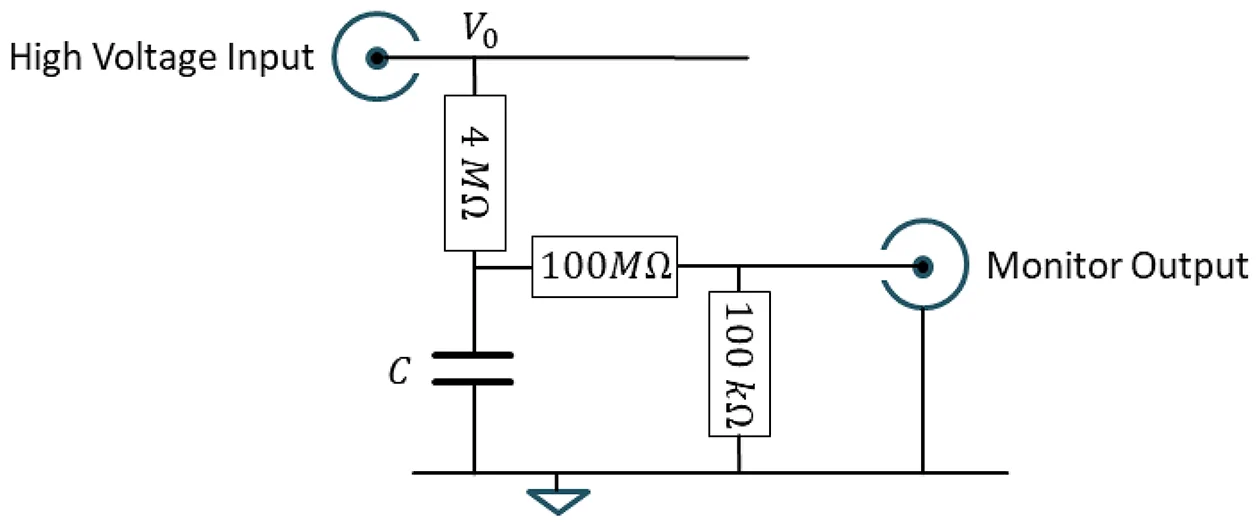
Basic circuit design of the capacitor BTM.

**Figure 2:**
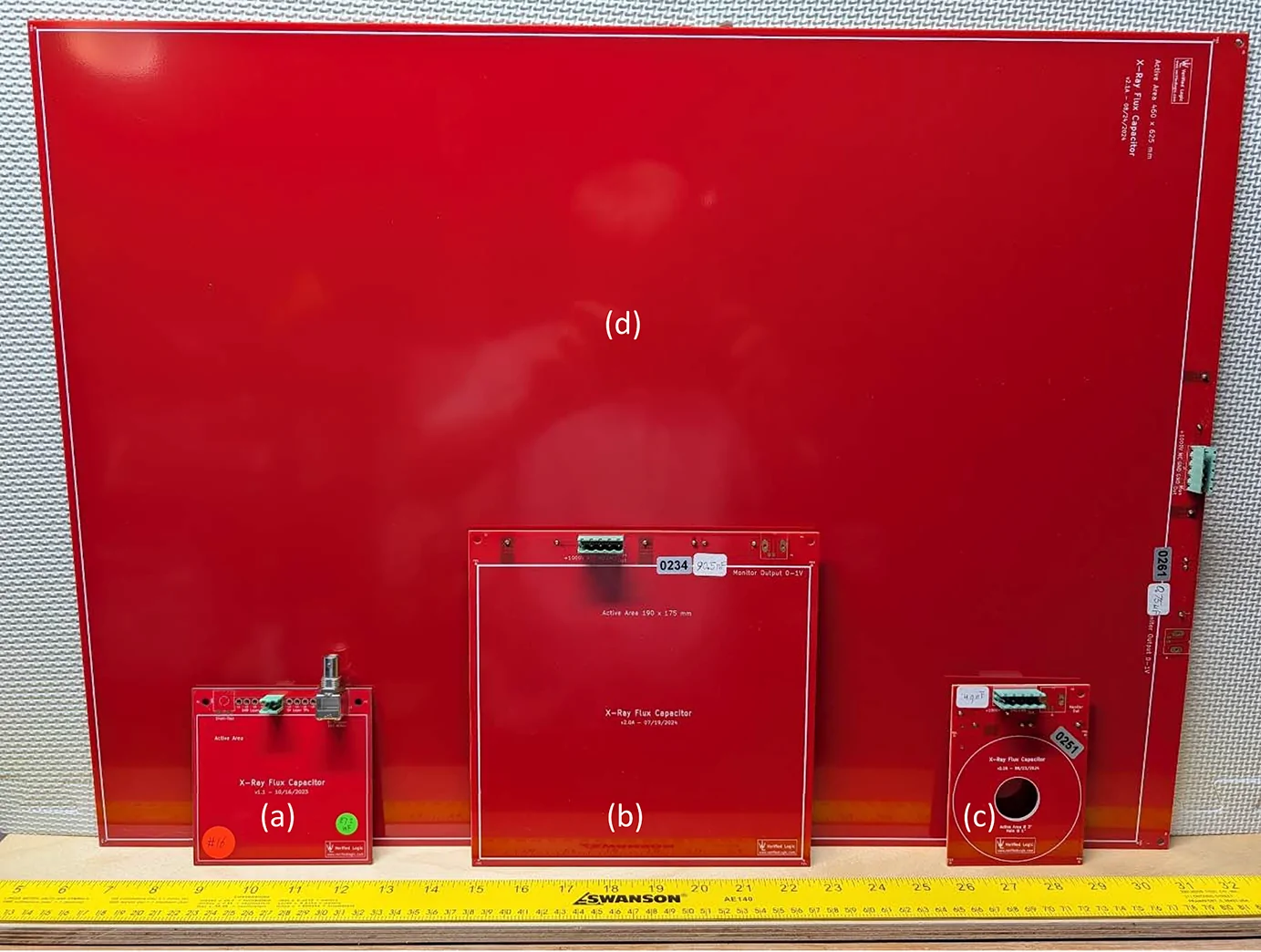
Photograph of the different prototype capacitor BTMs: (*a*) early prototype with sensitive area of 100 mm × 85 mm; (*b*) medium prototype with sensitive area 195 mm × 175 mm; (*c*) small prototype with 75 mm outer- and 25 mm inner diameter sensitive area; (*d*) large prototype with sensitive area 625 mm × 460 mm.

**Figure 3:**
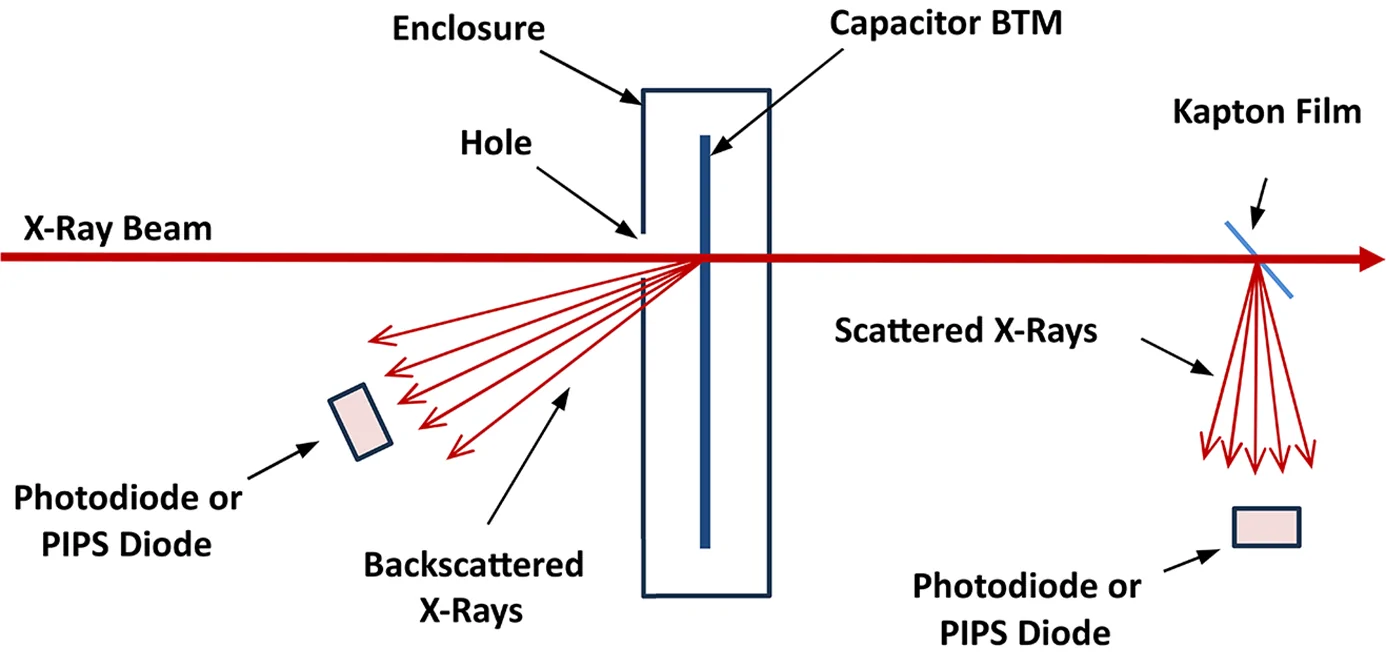
Schematic of typical setup at XCS or XPP.

**Figure 4:**
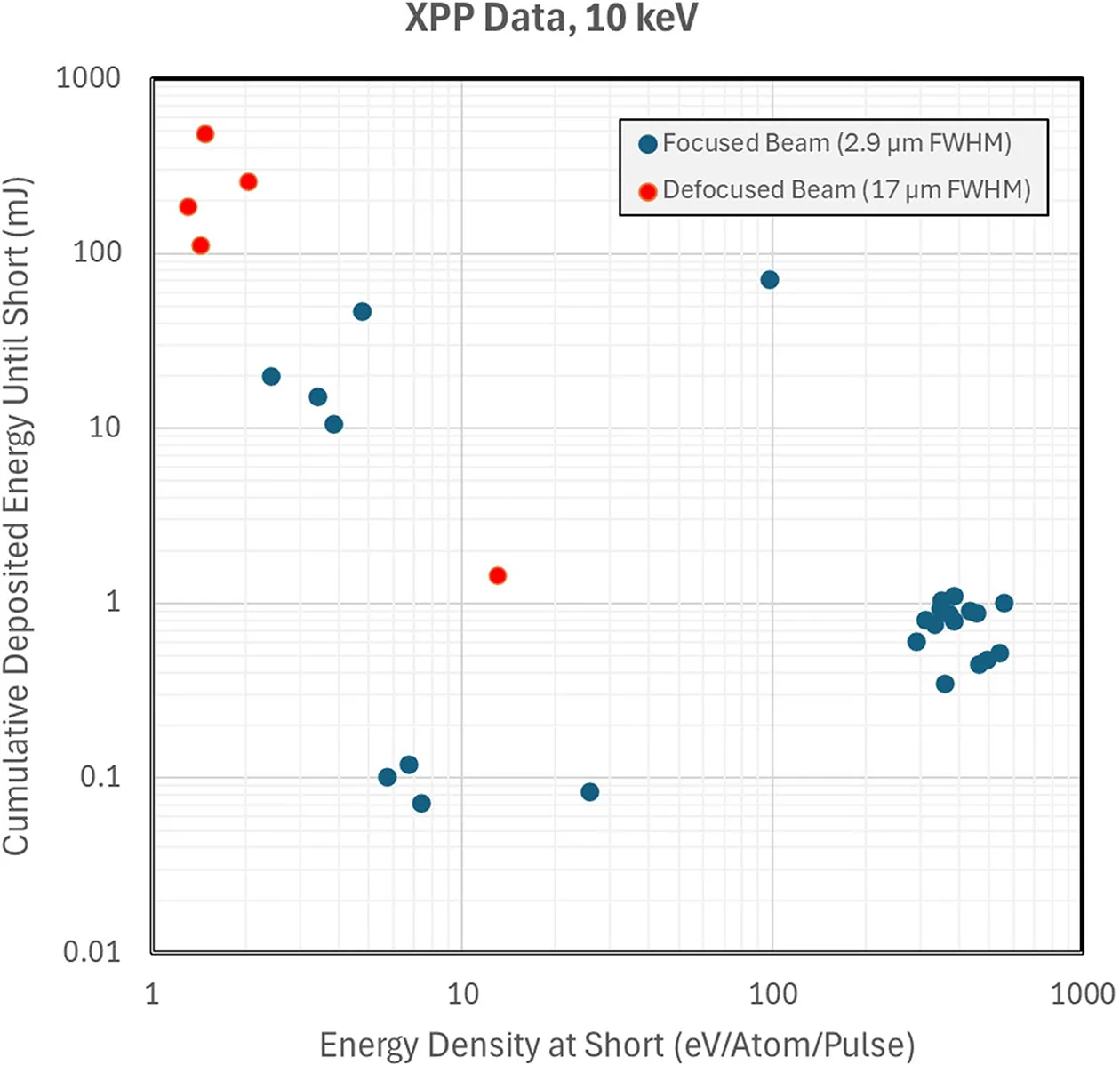
Cumulative energy deposited in the capacitor BTM until it shorted versus energy density at the time of the short for the 29 capacitors tested at XPP. Marker colors indicate whether the beam was focused or defocused, as shown in the legend.

**Figure 5:**
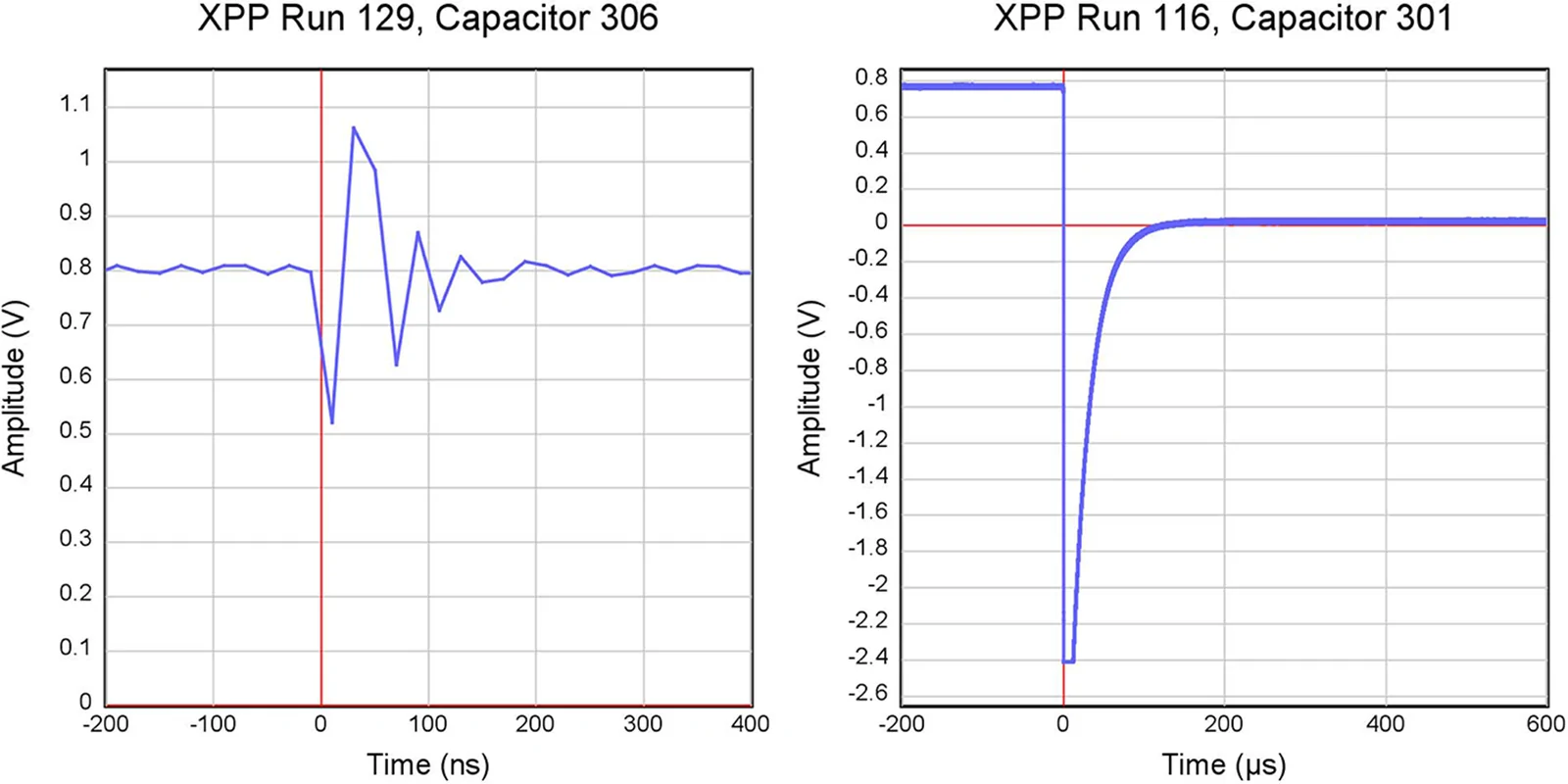
Fast traces collected with the oscilloscope. Left: the ‘blip’ that triggered the oscilloscope during run 129 at XPP. Right: oscilloscope signal of a more typical run.

**Figure 6:**
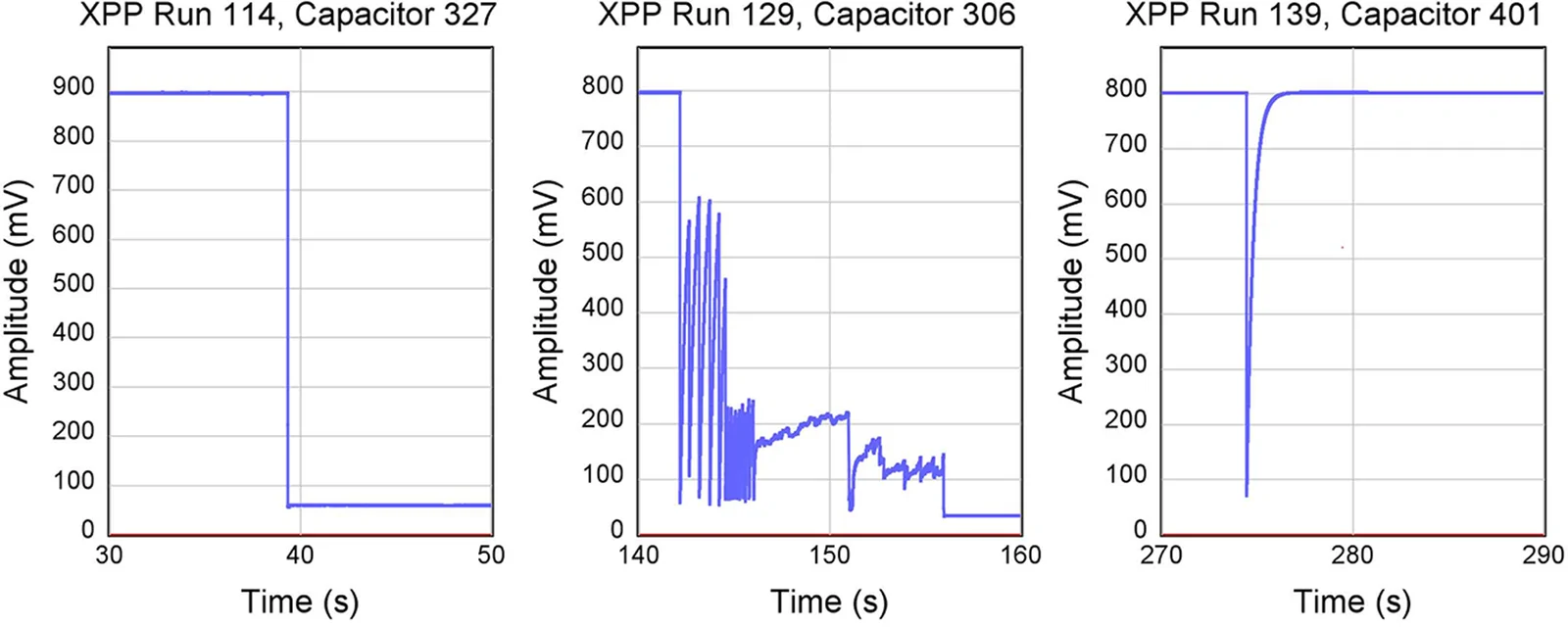
Examples of traces collected with the slow ADC. Left: device shorted and did not recover. Center: device shorted and attempted to recover but failed. Right: device shorted and self-healed.

**Figure 7:**
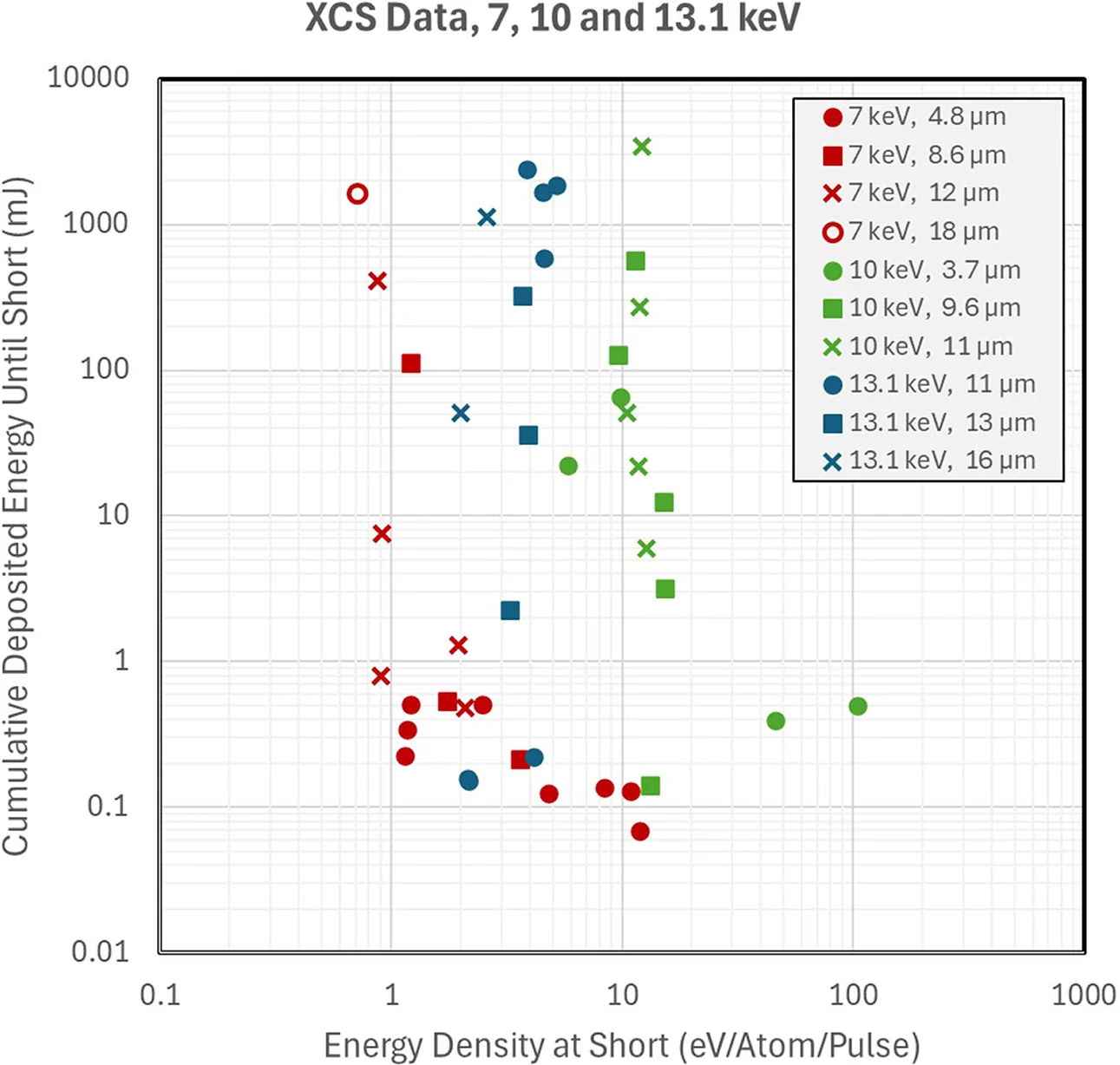
Cumulative energy deposited in the capacitor BTM until it shorted versus energy density at the time of the short for 40 capacitors tested at XCS at three X-ray energies: 13 keV (blue data points), 7 keV (red) and 10 keV (green). Marker type indicates the beam diameter (FWHM) in µm, as shown in the legend.

**Figure 8:**
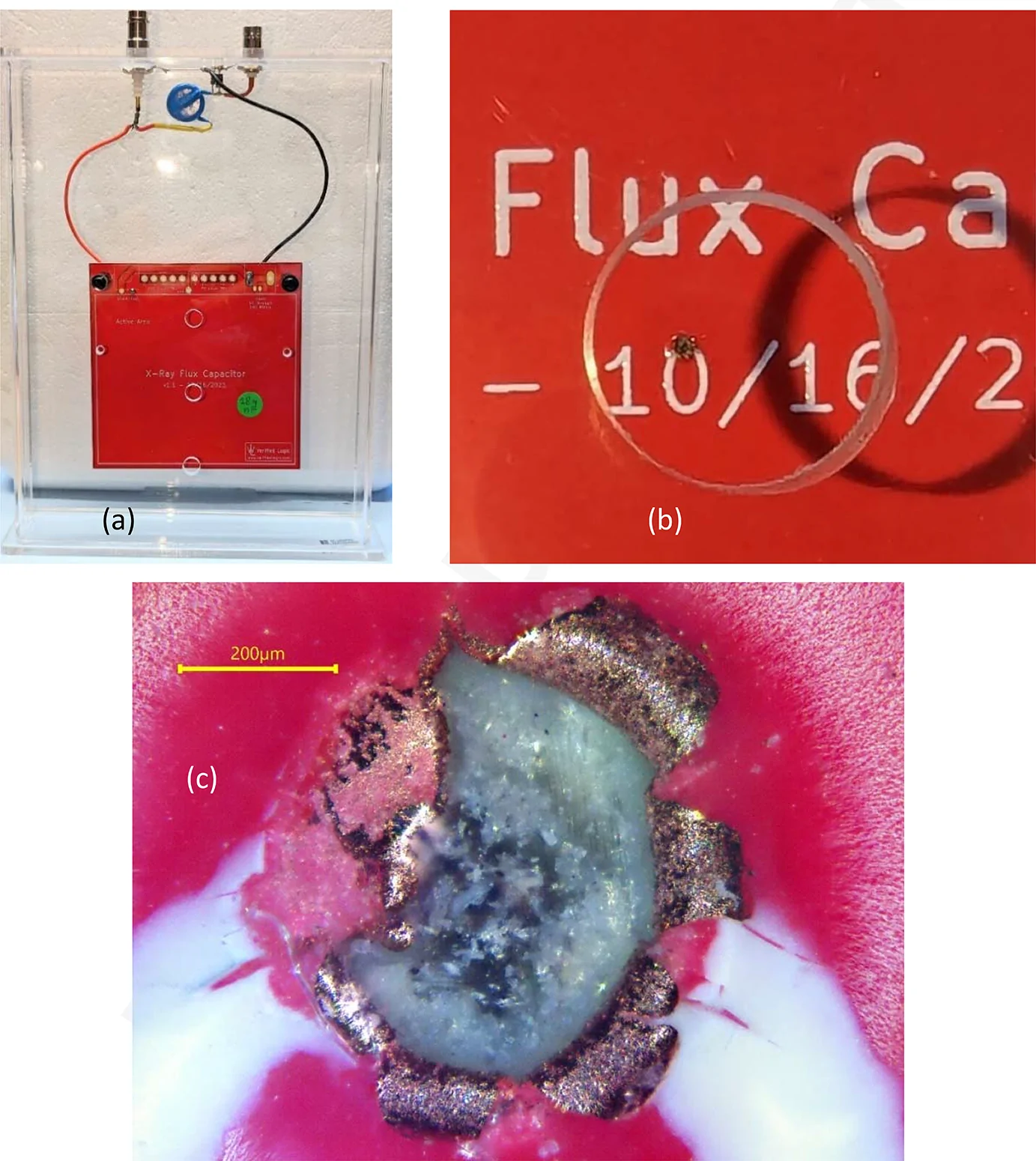
Photographs of the results of the short of the first high-voltage prototype capacitor BTM: (*a*) capacitor BTM in its enclosure; (*b*) zoom into the region where the beam struck the device; (*c*) microscope image made by focus-stacking several images acquired using a an Olympus BHM microscope with an OptixCam OCS-SK2-5.2X at 100× magnification.

**Figure 9:**
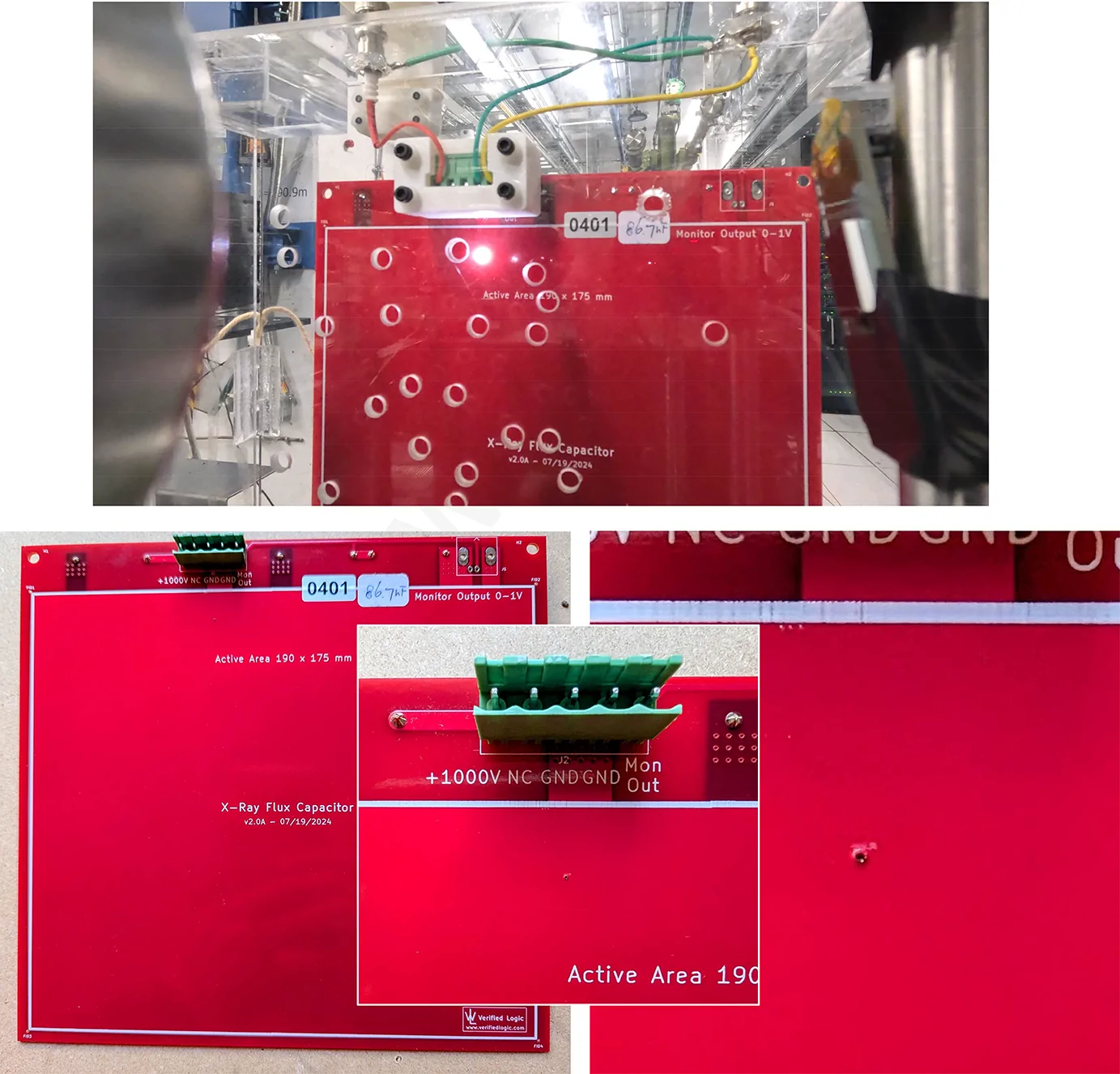
Top: the moment of the short in the beam at XPP for capacitor BTM 401. Bottom: zoomed in images of capacitor BTM 401 after the short. A burn mark is clearly visible.

**Figure 10:**
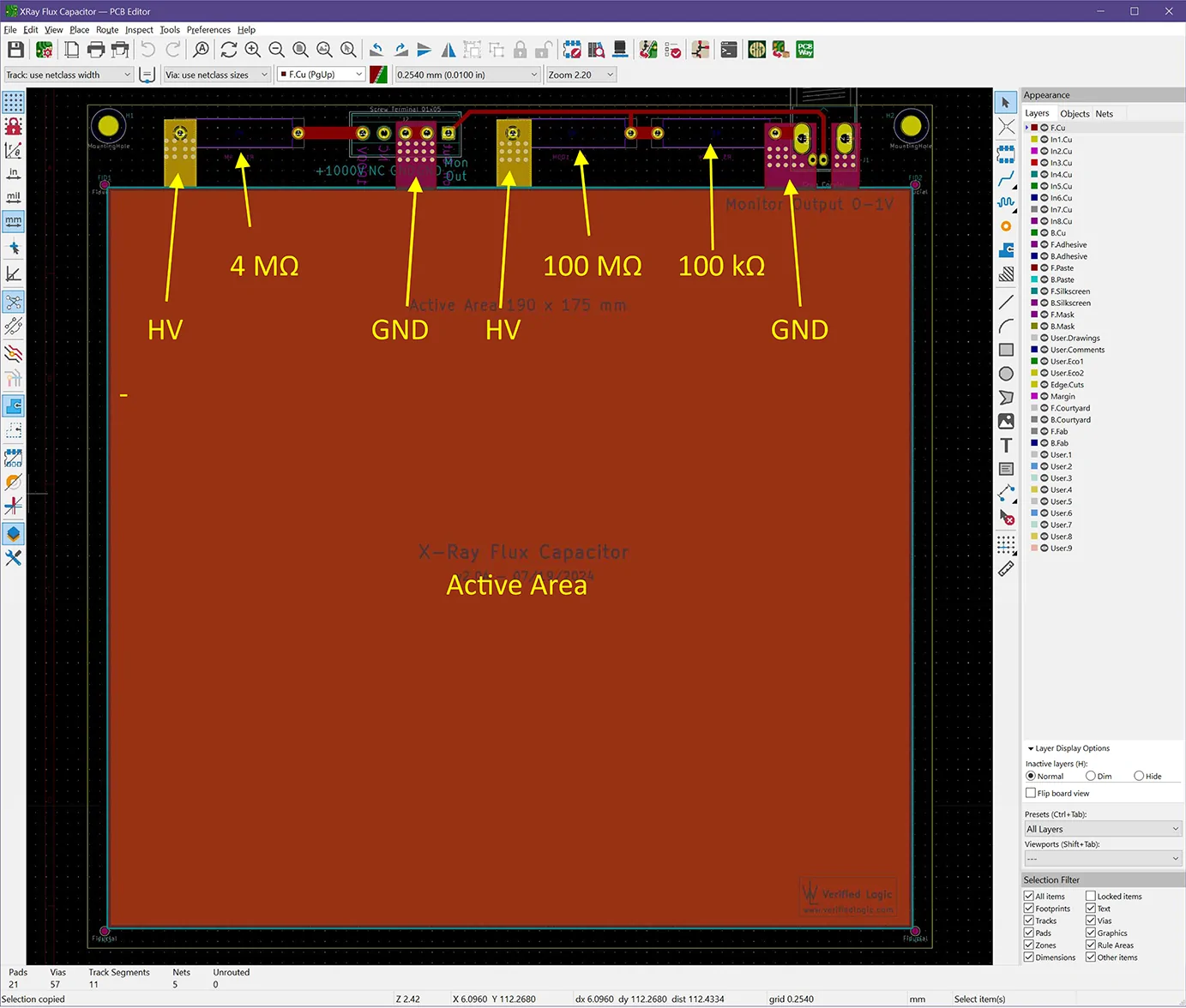
KiCAD board layout of the medium-sized capacitor BTM prototype. The active area in the layout is indicated in red.

**Figure 11:**
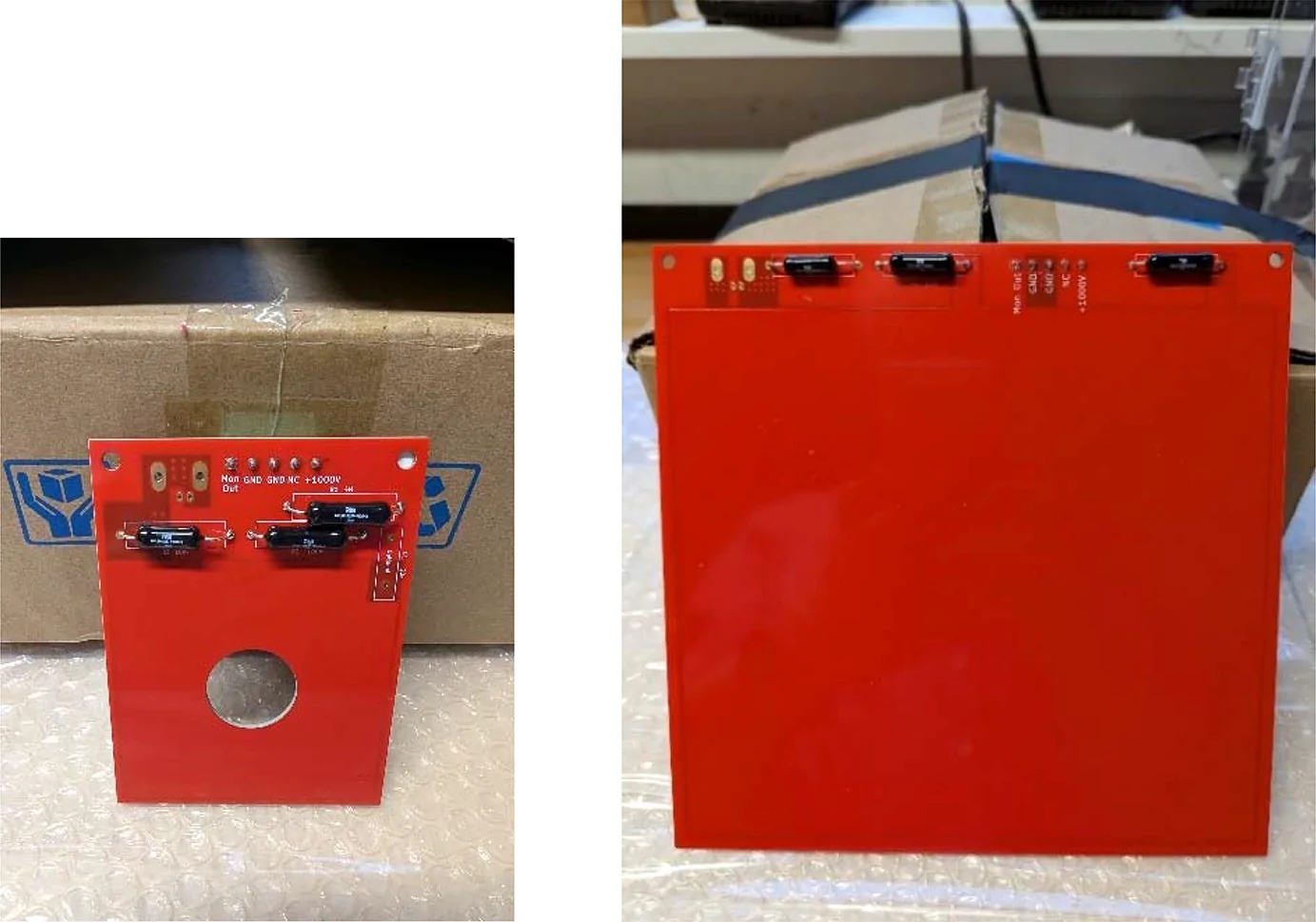
Resistors are mounted on the back of the PCB.

**Table 1:** Beam parameters used in the experiments

Instrument	X-ray energy (keV)	Beam size (FWHM) (µm)	Focused unattenuated pulse energy at IP (mJ pulse^−1^)	Lowest energy density at short (eV atom^−1^ pulse^−1^)
XCS	7.17	4.8–18	0.069	0.7
XCS	9.99	3.7–11	0.177	5.8
XCS	13.1	11–16	0.120	1.6
XPP	10.0	2.9–17	0.486	1.3

**Table 2:** Layer structure of the tested capacitor BTMs

Layer #	Name	Material	Thickness (mm)
0	Top solder mask and silkscreen	Unspecified resin	0.01
1	1st copper	Copper	0.035
2	1st dielectric	FR-4 (PrePreg)	0.1
3	2nd copper	Copper	0.035
4	2nd dielectric	FR-4 (Core)	0.1825
5	3rd copper	Copper	0.035
6	3rd dielectric	FR-4 (PrePreg)	0.1
7	4th copper	Copper	0.035
8	4th dielectric	FR-4 (Core)	0.1825
9	5th copper	Copper	0.035
10	5th dielectric	FR-4 (PrePreg)	0.1
11	6th copper	Copper	0.035
12	6th dielectric	FR-4 (Core)	0.1825
13	7th copper	Copper	0.035
14	7th dielectric	FR-4 (PrePreg)	0.1
15	8th copper	Copper	0.035
16	8th dielectric	FR-4 (Core)	0.1825
17	9th copper	Copper	0.035
18	9th dielectric	FR-4 (PrePreg)	0.1
19	10th copper	Copper	0.035
20	Bottom solder mask and silkscreen	Unspecified resin	0.01

## Data Availability

Some of the data may be available from the authors upon reasonable request.

## Appendix A Capacitor BTM PCB design

### A1. PCB design

The prototype design of the capacitor BTM consists of PCBs of various sizes. The PCB design of the 195 mm × 175 mm version is shown in Fig. 10 ▸.

The PCB has ten 1-ounce copper (35 µm thick) layers: five that are nominally charged to the design voltage of 1000 V, alternating with five ground layers. The front and back layers are covered with solder mask. The physical stack up is shown in Table 2 ▸. The solder pads at the top left of center in Fig. 10 ▸ are for a keyed five-pin header connector that provides the high voltage and allows for reading out the monitor voltage (1/1000 of the operating HV), see also Fig. 9 ▸ bottom-center. A BNC connector for reading out the monitor voltage can optionally be installed at the top right.

The three HV-rated resistors (see Fig. 10 ▸) are connected to the HV and ground planes using pads with vias, in such a way that it is not possible to read out a correct monitor voltage without the high voltage actually being present on the five layers that are charged to the design voltage. They are mounted on the back of the PCB, see for example Fig. 11 ▸.

The design is scalable to any size PCB available from PCB fabs and can be adapted to a large variety of shapes and configurations.

### A2. Fail-safe operation notes

There are some features of the capacitor BTM that help with constructing a fail-safe beam containment diagnostic by combining the device with safety-rated latching electronics. There is only one input voltage to the capacitor BTM: the high voltage connection. If it is disconnected, or if the high voltage power supply is turned off or there is a power failure, or if the high voltage cable is broken, the monitor output voltage will be low and cause the electronics to register a trip. If the monitor output is disconnected, or the monitor cable is broken, the monitor voltage measured by the electronics will be low and cause a trip. The read-out electronics module should be constructed such that a measured monitor voltage below a set threshold is interpreted as a trip. The module should latch this trip signal and present the trip condition as a low signal at a designated output that is connected to the Beam Containment System (BCS) and/or Personnel Protection System (PPS). This has the advantage that, if the readout electronics are turned off or there is a power failure on that side, the resulting voltage received by the BCS/PPS will be low (*e.g.* by using a pull-down resistor to ground), which the BCS/PPS should interpret as a fault condition. The readout electronics module should at all other times assert a high signal to signify no trip has occurred.
